# Glucose Injection Into Yolk Positively Modulates Intermediary Metabolism and Growth Performance in Juvenile Nile Tilapia (*Oreochromis niloticus*)

**DOI:** 10.3389/fphys.2020.00286

**Published:** 2020-04-17

**Authors:** Suksan Kumkhong, Lucie Marandel, Elisabeth Plagnes-Juan, Vincent Veron, Surintorn Boonanuntanasarn, Stephane Panserat

**Affiliations:** ^1^School of Animal Technology and Innovation, Institute of Agricultural Technology, Suranaree University of Technology, Nakhon Ratchasima, Thailand; ^2^INRAE, Université de Pau et des Pays de l’Adour, E2S UPPA, Nouméa, France

**Keywords:** nile tilapia, nutritional programming, glucose injection, gene expression, glucose metabolism

## Abstract

The aim of this study was to explore for the first time in omnivorous fish the concept of nutritional programming. A nutritional stimulus was accomplished by microinjecting 2 M glucose into yolk reserves during the alevin stage in Nile tilapia (*Oreochromis niloticus*). At the molecular level in fry, at 1 week post-injection, glucose stimuli were associated with the up-regulation of genes involved in glycolysis (*pklr*, *hk1*, *hk2*, and *pkma*), glucose transport (*glut4*) pathways and down-regulation of genes related to gluconeogenesis (*g6pca1*, *g6pca2*, and *pck1*) and amino acid catabolism (*asat*, *alat*) (*P* < 0.05), demonstrating that the larvae well received the glucose stimulus at a molecular level. Moreover, 20 weeks after glucose injection, early glucose stimuli were always linked to permanent effects in juvenile fish, as reflected by a higher level of glycolytic enzymes [*gck*, *hk1* and *hk2* at both mRNA and enzymatic levels and pyruvate kinase (PK) activity]. Finally, the effects of the glucose stimulus history were also examined in fish fed with two different dietary carbohydrate/protein levels (medium-carbohydrate diet, CHO-M; high-carbohydrate diet, CHO-H) in juvenile fish (during weeks 20–24). As expected, the CHO-H diet induced the expression of glycolytic and lipogenic genes (*gck*, *pklr*, *hk1*, *hk2*, *fpkma, fasn*, and *g6pd*) and suppressed the expression of gluconeogenic and amino acid catabolism genes (*g6pca1*, *pck1*, *pck2*, *asat*, *alat*, and *gdh*). Nevertheless, the early glucose stimulus led to persistent up-regulation of glycolytic enzymes (*gck*, *pklr*, *hk1*, and *hk2*) at both the mRNA and enzyme activity levels and glucose transporter *glut4* as well as lower gluconeogenic *pck1* gene expression (*P* < 0.05). More interestingly, the early glucose stimulus was associated with a better growth performance of juvenile fish irrespective of the diets. These permanent changes were associated with DNA hypomethylation in the liver and muscles, suggesting the existence of epigenetic mechanisms at the origin of programming. In conclusion, for the first time in tilapia, early glucose stimuli were found to be clearly associated with a positive metabolic programming effect later in life, improving the growth performance of the fish.

## Introduction

Fish nutrition is one of the most important perspectives for developing sustainable fish farming, which has become an important food-producing sector for global food security. In order to improve fish nutrition, it is scientifically challenging to not only search for potential alternative feed ingredients and feed supplementation but also perform research into understanding fish metabolism and how it can be modulated. Recently, several hypothesis-driven scientific approaches for fish nutrition have been demonstrated (Panserat et al., 2019). Nutritional programming is an issue that needs to be explored to examine how it modulates metabolism in fish (Geurden et al., 2007; Fang et al., 2014; Rocha et al., 2014, 2015). Metabolic programming is defined as the long-term consequences of environmental events or stimuli during early development that exert permanent effects on metabolism and physiology later in life (Lucas, 1998; Symonds et al., 2009). Persistence of long-term effects of environmental stimuli was proposed to be linked with epigenetics, which might be transmissible from one cell generation to another (Gavery and Roberts, 2017; Panserat et al., 2017; Veron et al., 2018). The concept of nutritional programming might offer a potential application in fish nutrition for the goal of modifying fish metabolism for efficient use of alternative feed, such as plant-based ingredients, high-carbohydrate diet and low-fish-oil/fishmeal-containing diet (Geurden et al., 2013; Rocha et al., 2014, 2015; Lazzarotto et al., 2016; Clarkson et al., 2017). In order to test whether nutritional programming exists, several factors should be explored, including the types of the early environmental/nutritional stimuli, testing of different developmental windows for applying the stimulus and the types of challenges during adult stage (Geurden et al., 2007, 2014; Mennigen et al., 2013; Fang et al., 2014; Rocha et al., 2014, 2015, 2016a,b; Gong et al., 2015; Marandel et al., 2016a,b).

In aquaculture, digestible dietary carbohydrates are among the less expensive sources of energy, and they are generally incorporated in fish diets as much as possible to produce low-cost aquafeed. As a better understanding of nutritional regulation of carbohydrate metabolism in fish would enable the efficient use of carbohydrates as an energy source to spare proteins for growth, nutritional regulation of glucose metabolism has been intensively studied in various fish species in the last decades (Panserat et al., 2000, 2001, 2009; Polakof et al., 2012; Marandel et al., 2015; Kamalam et al., 2017; Seiliez et al., 2017; Boonanuntanasarn et al., 2018a, b). In order to improve the metabolic use of carbohydrates in carnivorous fish, generally known to be poor users of this nutrient, the concept of nutritional programming for carbohydrate metabolism has recently been demonstrated in rainbow trouts (Geurden et al., 2007, 2014; Mennigen et al., 2013; Marandel et al., 2016a, b), sturgeon (Gong et al., 2015) and gilthead seabream (Rocha et al., 2016a, b). However, data on nutritional programming in omnivorous and herbivorous fish, known to be good carbohydrate users, are limited to model fish species such as zebrafish (Ribas and Piferrer, 2014). Therefore, testing the nutritional programming of glucose metabolism in omnivorous and herbivorous fish would provide, for the first time, scientific evidence for comparative studies among fish species.

Nile tilapia (*Oreochromis niloticus*) is an omnivorous fish species that can use high levels of dietary carbohydrates (Shiau and Peng, 1993; Wang et al., 2005; Kamalam et al., 2017). Our previous findings unambiguously demonstrated adapted molecular responses of glucose metabolism to carbohydrate intake even during a long-term feeding trial (Boonanuntanasarn et al., 2018a, b). Indeed, high-dietary-carbohydrate intake led to increased hepatic glycogen and muscle glycolysis (higher PK enzyme expression) and was associated with reduced gluconeogenic capacities (lower expression of genes for glucose-6-phosphatase and phosphoenolpyruvate carboxykinase enzymes). Hence, investigating the potential effects of nutritional programming on glucose metabolism in Nile tilapia would expand our knowledge of carbohydrate nutrition in Nile tilapia, which is the second most cultured fish after the common carp.

In the present study, nutritional programming of glucose metabolism in tilapia was achieved using an early glucose stimulus in the alevin stage. Glucose was microinjected into sac-fry larvae, and the effects of the glucose stimuli on the growth performance in alevins and juvenile fish were determined. In addition, molecular and enzymatic analyses of hepatic and muscle enzymes of glucose metabolism, plasma metabolites and tissue compositions were performed in juvenile fish before and after a final dietary challenge with a high-carbohydrate diet (67%) compared to a medium-carbohydrate diet (37%). Finally, because one of the main possible mechanisms underlying programming effects is epigenetics (Marandel et al., 2016a; Panserat et al., 2017), we also analyzed the global DNA methylation in the liver and muscles of fish depending on the glucose (injection) history and high-carbohydrate challenging diets.

## Materials and Methods

### Fish for the Experiment, Experimental Design and Diet Formulation

**FIGURE 1:**
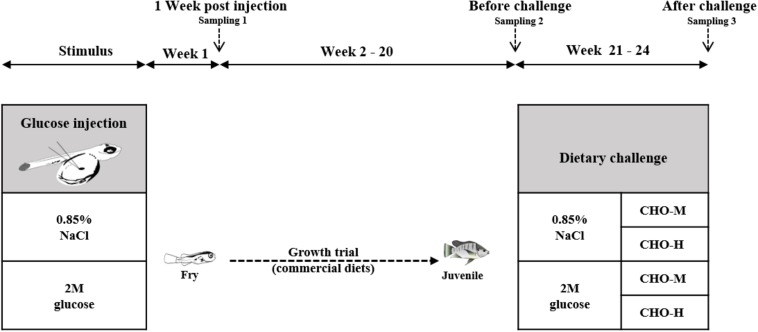
Experimental plan of glucose stimulus by injection (history) and dietary carbohydrate challenge test. Nile tilapia alevins were injected with either a saline (0.85% NaCl) or a glucose (2 M) solution into the yolk sac at the newly hatching stage. At 1 week post injection (wpi), larvae were sampled to determine the body glucose and glycogen contents and the expression of genes that are involved in carbohydrate metabolism. See Table 1 for the list of genes and their respective primers. Subsequently, the fish were cultured for a growth trial during 2–20 wpi. During the growth trial, commercial diets (during 2–8 wpi: 40% CP + 8% CF; during 9–20 wpi: 32% CP + 4% CF) were used. During 20–24 wpi, the fish were subjected to a challenge test with different dietary carbohydrate levels (37% carbohydrates, CHO-M; 67% carbohydrates, CHO-H). Fish sampling was carried out before (20 wpi) and after (24 wpi) the challenge test.

All experimental protocols were approved by the Ethics Committee of Suranaree University of Technology, Animal Care and Use Committee (Approval no. A-18/2562). The Nile tilapia alevins used in this study were obtained from broodstock obtained from a farm at Suranaree University of Technology, Nakhon Ratchasima, Thailand. Nile tilapia broodstock (0.8–1.2 kg) were cultured in an earthen pond (800 m^2^) and fed with a commercial feed (30% crude protein (CP) + 4% crude fat (CF) at 3% body weight) at 9:00 and 16:30 daily. A schematic view of the experimental design is shown in Figure 1. In order to investigate the effects of glucose microinjection on the survival rate and glucose content, the experimental design was completely randomized with three treatments – non-injected control, injection of a solution of 0.85% NaCl (saline) and injection of a solution of 2 M glucose – on six replicates (families). To investigate the effects of glucose stimuli on carbohydrate metabolism in the long term, two groups of fish (six replicates) from either the saline (0.85%) or the 2 M glucose injection group were reared for 20 weeks. For the dietary challenge, a 2 × 2 factorial design with the two stimuli (0.85% NaCl and 2 M glucose) combined with two dietary carbohydrate levels (37% CHO-M and 67% CHO-H) was employed in a completely randomized design using six replicates (cages). Table 1 presents the ingredients of the two diets and the proximate composition of the two experimental diets [i.e., the medium-carbohydrate diet (CHO-M) and the high-carbohydrate diet (CHO-H)]. The diets were analyzed for moisture, CP, CF, and ash content according to the standard method of the Association of Official Analytical Chemists [AOAC] (1990).

**TABLE 1:** Ingredients and chemical compositions (g kg^–1^) of the challenging diets.

Ingredients	CHO-M	CHO-H
Fish meal	350	140
Soybean meal	300	60
Rice flour	150	700
Rice bran	180	30
Soybean oil	—	40
Dicalcium phosphate	—	10
Fish premix^a^	20	20
**Proximate composition (g kg*^–^*^1^ dry weight)**		
Dry matter	957.7	957.3
Protein	356.7	154.9
Fat	69.0	64.8
Fiber	28.9	8.6
Ash	129.2	60.3
NFE^b^	373.9	668.8
Gross energy (kJ g^–1^)	17.6	17.2

*^a^Vitamin and trace mineral mix provided the following (IU kg^–1^ or g kg^–1^ diet): biotin, 0.25 g; folic acid, 0.003 g; inositol, 0.25 mg; niacin, 0.0215 g; pantothenic acid, 0.03 g; vitamin A, 5,000 IU; vitamin B1, 0.0025 g; vitamin B2, 0.0012 g; vitamin B6, 0.0075 g; vitamin B12, 0.00005 mg; vitamin C, 1 g; vitamin D3, 1,000 IU; vitamin E, 100 IU; vitamin K, 0.008 g; copper, 0.02 g; iron, 0.2 g; selenium, 0.3 mg; zinc, 0.32 g. ^b^Nitrogen-free extract = dry matter - (CP + crude lipid + crude fiber + ash).*

### Alevin Collection and Microinjection Protocol and Fish Culture

A schematic view of the feeding plan is shown in Figure 1. Fertilized eggs at stages 11–12 (Fujimura and Okada, 2007) were collected from female Nile tilapia (six replications) and transferred to a hatching tray (20 × 30 × 5 cm^3^) with flow-through freshwater at 27–29°C. After 3 days, the hatched larvae were selected for microinjection. For normal-saline-injected and glucose-stimulated alevins (100 larvae/replication), 0.85% NaCl and 2M glucose (in 0.85% NaCl), respectively, were micro-injected into the yolk sac of the newly hatched larvae (stage 17) (Fujimura and Okada, 2007). A 0.5 mm diameter glass capillary (EG-400; Narishige, Tokyo, Japan) was used to make a needle to deliver 60 nL of normal saline and 2M glucose into the yolk sac of the newly hatched larvae (3–10 h after hatching). After injection, each group of microinjected larvae were transferred to a hatching tray (20 × 30 × 5 cm^3^) and reared with gentle aeration for 1 week. Non-injected control larvae were also reared to compare their survival rates at 1 week after injection (week post-injection, wpi). The glucose levels in the yolk reserves of all the experimental groups were determined. For that measure, the yolk sacs of 10 larvae were removed, pooled together, weighed and homogenized. The yolk homogenates were centrifuged at 3,000 × g at 4°C for 5 min. The aqueous phase (the layer between the lipid and cell debris) was then transferred to a new tube. An equal volume of de-ionized water was mixed and used for glucose determination in accordance with Trinder’s method (Trinder, 1969).

Experimental larvae (normal-saline-injected and glucose-stimulated alevins) were transferred to cages (40 × 40 × 60 cm^3^) with aeration (six replicates). In this study, only male tilapia were used to avoid confounding effects linked to sex. During weeks 1–4, all experimental larvae were fed with a commercial powder feed (40% CP + 8% CF) mixed with 17α-methyltestosterone at 60 mg kg^–1^ five times daily (at 09:00, 11:00, 13:00, 15:00, and 17:00) (Boonanuntanasarn et al., 2018a). Subsequently, during weeks 5–20, the fish were transferred to cement ponds (2 × 2 × 0.8 m^3^) and fed with a commercial feed (32% CP + 4% CF) *ad libitum* twice daily (at 09:00 and 16:00). Fish mortality was monitored daily. In order to assess the growth performance, the fish were weighed and the feed intake was recorded every 4 weeks throughout the experimental period. Air and water temperatures were measured daily and were 20.6–32.0°C and 18.8–27.6°C, respectively. The dissolved oxygen (DO) content and pH were measured once a week using a DO meter and a pH meter, and the values were within acceptable ranges of 4.2–5.8 mg L^–1^ and 7.5–7.8, respectively.

During weeks 20–24, 10 fish from each replicate were randomly selected and subjected to a dietary challenge with two different levels of dietary carbohydrates (CHO-M and CHO-H; Table 1). The fish (*n* = 10/replication, six replications) were then transferred to culture in a cage (90 × 80 × 110 cm^3^) for 4 weeks. Throughout the trial, the fish were fed twice daily (at 09:00 and 15:00). Fish mortality was monitored daily, and the growth performance was also recorded. Daily air and water temperatures were in range of 19.0–25.4°C and 16.4–23.0°C, respectively. The DO and pH were measured weekly, and their values were within acceptable ranges of 4.1–4.2 mg L^–1^ and 7.5–7.9, respectively.

### Fish Sampling and Blood Collection

At week 1, pooled larvae (pool of three whole-body larvae/replicate tank) were randomly selected and immediately frozen in liquid nitrogen and stored at −80°C for RNA extraction. At week 20, 5 h after feeding (i.e., the peak of glucose absorption in tilapia), five fish per tank were also sampled for plasma metabolites and chemical composition of the liver and muscles. Following anaesthesia, blood samples were collected from the caudal vein using a hypodermic syringe and mixed with K_2_EDTA (at 1.5 mg mL^–1^). Plasma was collected after centrifugation at 3,000 × *g* for 10 min at 4°C and stored at −80°C for metabolite analysis. Then, liver and muscle tissue samples were collected and frozen with liquid nitrogen and kept at −80°C for chemical composition analysis according to the AOAC. At week 24, fish sampling was performed as previously described for week 20.

### Blood Metabolite Analysis

Plasma metabolites were analyzed with 30 fish per experimental group (five fish/replication, *n* = 30 per experimental group). Plasma glucose was analyzed according to the GOD-PAP method (Trinder, 1969). The triglyceride levels were determined using the glycerol-3-phosphate oxidase-sodium *N*-ethyl-*N*-(3-sulfopropyl)-*m*-anisidine method as described by Bucolo and David (1973). Blood urea nitrogen (BUN) content was measured using a modified indophenol colorimetric method (Weatherburn, 1967).

### Total RNA Extraction and Relative Quantification of mRNAs

Relative gene expression was determined using quantitative real-time reverse transcription polymerase chain reaction (RT-PCR) of RNA extracted from whole-body larvae (week 1) (pool of three fish/replication; *n* = 18 per experimental group) and the liver and muscles (weeks 20 and 24) (two fish/replication; *n* = 12 per experimental group). Total RNA was extracted from tissue samples (week 1, pool of three larvae; weeks 20 and 24; 50 mg of liver and 100 mg of muscles) using a TRIzol reagent (Invitrogen, Carlsbad, CA, United States), according to the manufacturer’s instructions. Total RNA was quantified by NanoDrop (Thermo Fisher Scientific, Madison, WI, United States), and the quality was verified on 1% agarose gel. A SuperScript III RNase H Reverse-Transcriptase Kit (Invitrogen) with random primers (Promega, Charbonnières, France) was used, following the manufacturer’s protocol, to synthesize cDNA (*n* = 12 for each treatment group). A sample of 1 μg of total RNA was used for cDNA synthesis, using 100 units of SuperScript III enzyme and 40 units of RNase OUT enzyme. Reverse transcription was carried out in duplicate for each sample.

Supplementary Table S1 details the primer sequences used in the real-time RT-PCR assays (Yang et al., 2013; Boonanuntanasarn et al., 2018a, b). Glucose metabolic gene expression in the liver was analyzed, including glycolysis [glucokinase (GK), *gck*; phosphofructokinase, *pfklr*; pyruvate kinase (PK), *pklr*] and gluconeogenesis (glucose-6-phosphatase, g*6pca1*, and *g6pca2*; phosphoenolpyruvate carboxykinase cytosolic, *pck*1; mitochondria, *pck2*). Glucose use in muscles was analyzed by measuring the mRNA levels of glucose transporter (*glut4*) and glycolysis (hexokinase [HK] I/II, *hk1*, and *hk2*; phosphofructokinase, *pfkma* and *pfkmb*; PK, *pkma*). Lipogenic capacities (fatty acid synthase, *fasn*; glucose-6-phosphate dehydrogenase, *g*6*pd*) were examined. In addition, the enzymes involved in amino acid catabolism (glutamate dehydrogenase, *gdh*; alanine aminotransferase, *alat*; aspartate amino transferase, *asat*) were measured. A Roche LightCycler 480 system was used (Roche Diagnostics, Neuilly-sur-Seine, France) for real-time RT-PCR assays of transcripts of metabolic genes. Assays were performed using a reaction mix of 6 μL per sample, each of which contained 2 μL of diluted cDNA template (1:40), 0.24 μL of each primer (10 μM), 3 μL of LightCycler 480 SYBR^®^ Green I Master Mix (Roche Diagnostics) and 0.76 μL of DNase/RNase-free water (5 Prime GmbH, Hamburg, Germany). The PCR protocol was initiated at 95°C for 10 min for the initial denaturation of the cDNA and hot-start Taq polymerase activation, followed by 45 cycles of a three-step amplification program [15 s at 95°C, 10 s at 60–64°C (according to the primer set used) and 15 s at 72°C to extend the DNA]. The melting curves were systematically monitored (temperature gradient at 1.1°C/s from 65 to 97°C, five acquisitions/1°C) at the end of the last amplification cycle to confirm the specificity of the amplification reaction. Each PCR assay included replicate samples (duplicates of RT and PCR amplification, respectively) and negative controls (reverse-transcriptase- and cDNA-template-free samples, respectively). For the analysis of the mRNA levels, relative quantification of target gene expression was performed using the Roche Applied Science E-Method Pfaffl (2001). The relative gene expression of *ef*1α was used for the normalization of the measured mRNA in each tissue, as its relative expression did not significantly change over the sampling process (data not shown). In all cases, PCR efficiency was measured from the slope of a standard curve using serial dilutions of cDNA. In all cases, the PCR efficiency values ranged between 1.8 and 2.0.

### Chemical Composition and Glycogen Analysis

At weeks 20 and 24, chemical composition, including protein, fat and ash of the livers and muscles (five fish/replication, *n* = 30 per experimental group), was assessed according to Association of Official Analytical Chemists [AOAC] (1990). The livers (100 mg) and muscles (200 mg) were analyzed for glycogen (two fish/replication, *n* = 12 per experimental group). The glycogen content was determined using a hydrolysis technique that was previously described by Good et al. (1933). Each sample was ground in 1 M HCl (VWR, United States). An aliquot was saved at this stage and neutralized by 5 M KOH (VWR) to measure the free glucose content in the samples. After 10 min of centrifugation at 10,000 × *g* at 4°C, free glucose was measured using a plasma glucose kit (Glucose RTU; bioMérieux, Marcy-l’Étoile, France) according to the manufacturer’s instructions. The remaining ground tissue was boiled at 100°C for 2.5 h and then neutralized by 5 M KOH (VWR). After 10 min of centrifugation at 10,000 × *g* at 4°C, total glucose (free glucose + glucose obtained from glycogen hydrolysis) was measured using the same kit as before. The glycogen content was evaluated by subtracting free glucose levels.

### Enzymatic Assays

At weeks 20 and 24, muscles (200 mg) or livers (100 mg) were used to analyze enzyme activities. Tissue samples (from two fish/replication, total number of samples: 12) were homogenized in seven volumes of ice-cold buffer at pH 7.4 (50 mmol L^–1^ Tris, 5 mmol L^–1^ ethylenediaminetetraacetic acid [EDTA] and 2 mmol L^–1^ DTT) and a protease inhibitor cocktail (P2714; Sigma-Aldrich, St. Louis, MO, United States) and centrifuged for 10 min at 900 × *g* at 4°C. Assays for GK (EC 2.7.1.2) and HK (EC 2.7.1.1) activity were performed on the recovered supernatants. For PK (EC 2.7.1.40) activity, additional centrifugation was performed (20 min at 10,000 × *g* at 4°C), and the recovered supernatants were used for enzyme assays. The GK (high-*K*_*M*_) and HK (low-*K*_*M*_) enzymes were analyzed as described by Panserat et al. (2000). The activity of the PK enzyme was also measured as previously described by Panserat et al. (2001). Each enzyme activity was measured in duplicate at 37°C following the variation of absorbance of nicotinamide adenine dinucleotide phosphate (NADP^+^) at 340 nm. The reactions were started by the addition of a specific substrate; a PowerWave X (BioTek Instruments Winooski, VT, United States). De-ionized water was used as a blank for each sample. Enzyme activity units, defined as micro-moles of substrate converted into product per minute at the assay temperature, were expressed per milligram of protein. Protein concentration was measured in duplicate, according to Bradford (1976), using a protein assay kit (Bio-Rad, Munich, Germany) with bovine serum albumin as a standard.

### Analysis of Global DNA Methylation

For genomic DNA extraction, livers (50 mg) and muscles (100 mg) were digested in 1 mL of ice-cold extraction buffer (125 mM NaCl, 10 mM EDTA, 0.5% sodium dodecyl sulfate, 4 M urea and 10 mM Tris-HCl, pH 8.0) with 20 μg mL^–1^ proteinase K (P6556; Sigma-Aldrich), followed by incubation overnight at 37°C under agitation (250 rpm). Six replicates per condition were performed. After overnight digestion, an equal volume of phenol chloroform isoamyl alcohol (25:24:1) was added to each sample and mixed by inverting tubes. Samples were then centrifuged for 15 min at 10,000 × *g* at room temperature. Aqueous phases were then transferred into new tubes, and then 0.25 volume of 5 M NaCl was added, followed by two volumes of ice-cold absolute ethanol, and then incubated at −20°C for 15 min. After centrifugation at 10,000 × *g* at 4°C for 15 min, DNA pellets were obtained, washed with 1 mL of 75% ice-cold ethanol and then re-centrifuged for 15 min at 10,000 × *g* at 4°C. The pellets were then dried and re-suspended in 100 μL of DNase-free water. Samples were submitted to RNase treatment using 1 μg of RNase A (R4642; Sigma-Aldrich) and incubated for 1.30 h at 37°C under stirring (250 rpm). The quality of the genomic DNA was checked on 1% agarose gel, and quantification was carried out using NanoDrop (Thermo Fisher). The DNA global methylation pattern (C^m^CGG methylation pattern) was analyzed using the LUminometric Methylation Assay (LUMA) according to Karimi et al. (2006).

### Data Analysis

All data were analyzed using SPSS for Windows, version 12 (SPSS Inc., Chicago, IL, United States).

In order to determine the effects of glucose microinjection on the survival rate and glucose content, one-way analysis of variance (ANOVA) was performed. When significant differences were found among the groups, Tukey’s method was used to rank the groups. In addition, an independent *t*-test analysis was conducted to evaluate the differences between the two groups at weeks 1 and 20: saline (0.85% NaCl) versus glucose (2 M glucose) stimuli. After the nutritional challenge (week 24), the statistical factors consisted of the analysis of the effects of the glucose stimulus history, dietary carbohydrate level and their interactions. Two-way factorial ANOVA was also carried out. When the interaction of the factors was statistically significant, one-way ANOVA following Tukey’s range test was performed to rank the treatment combination groups. Throughout the experiment, the effects and differences were declared to be significant when *P* < 0.05.

## Results

### Direct Effects of Early Glucose Stimuli on Larval Survival and Expression of Glucose Metabolic Genes

**FIGURE 2:**
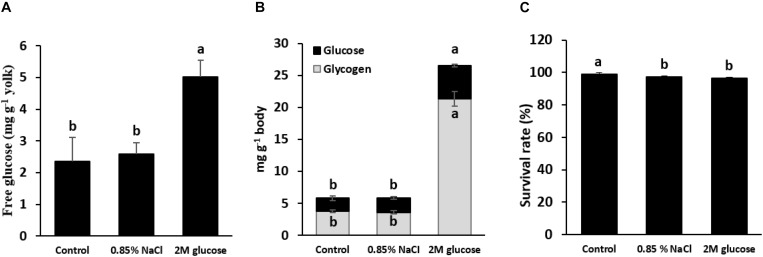
Glucose content in the yolk reserves of alevins and their subsequent bodily glucose and glycogen content. **(A)** Glucose levels in the yolk of alevins that were microinjected with saline (0.85% NaCl) or glucose (2 M), as well as the control (non-injected alevin). Note that fish alevins were sampled for examination of the glucose level in the yolk sac immediately after injection. At 1 wpi, the glucose content and glycogen content were examined in the bodies of larvae injected with saline (0.85% NaCl) and glucose (2 M) and in the control (non-injected) larvae **(B)**, and their survival rates were determined **(C)**. Different letters in the bar graph indicate significant differences (*P* < 0.05).

Early glucose stimuli were performed using a microinjection of either 2 M glucose or normal saline (0.85% NaCl) into the yolk sac of the alevins. Before glucose injection, the glucose levels in the yolk reserves of the control (non-injected fish) and normal-saline-injected yolks were 2.4–2.6 mg g^–1^ yolk. Glucose injection significantly increased the level of glucose in the yolk to 4.1–5.6 mg g^–1^ yolk (Figure 2A), indicating that injecting 2 M glucose is effective to overload the glucose content in yolk. Figure 2B demonstrated that injection 2 M glucose led to an increase in not only the glucose level, but also the glycogen content in fish larvae at 1 wpi. The survival rates of the control, saline-injected and glucose-injected larvae at 1 wpi are shown in Figure 2C. Although microinjection slightly reduced the survival rates of tilapia larvae, there were no significant differences in the survival rates between normal-saline-injected and glucose-injected larvae (*P* < 0.05), indicating that this level of glucose stimulus does not cause detectable detrimental effects on fish. Note that no malformities were observed in any fish throughout the experimental period. As the yolk was completely absorbed one week after hatching, the effects of glucose stimuli on the expression of genes related to glucose metabolism were determined in larvae at 1 wpi. Our results showed significant up-regulation of genes involved in glycolysis in the liver and muscles, including hepatic *pklr* and muscular *hk1* and *hk2* (*P* < 0.05) (Table 2). In addition, the *glut4* mRNA level was significantly increased in glucose-injected larvae. Moreover, down-regulation of gluconeogenesis and amino acid catabolism was observed for *g6pca1*, *g6pca2*, *pck1*, *asat*, and *alat* genes (*P* < 0.05). It can be noted that there were no significant differences in the transcripts of any detected genes related to lipogenesis (*P* > 0.05). Overall, glucose stimuli showed direct significant effects on glucose metabolic pathways.

**TABLE 2:** mRNA levels of genes involved in carbohydrate metabolism in the whole body of Nile tilapia (at 1 wpi) that were microinjected with either 0.85% NaCl or 2 M glucose (mean ± SD, *n* = 6).

Gene	0.85% NaCl	2 M glucose	*P*-value
**Glycolysis**			
*gck*	0.8 ± 0.3	1.5 ± 0.8	0.067
*pfklr*	1.1 ± 0.2	0.9 ± 0.3	0.156
*pklr*	0.7 ± 0.2	1.0 ± 0.3	0.030
**Gluconeogenesis**			
*g6pca1*	1.3 ± 0.1	0.7 ± 0.1	<0.001
*g6pca2*	1.3 ± 0.1	0.5 ± 0.1	<0.001
*pck1*	1.3 ± 0.1	0.6 ± 0.2	<0.001
*pck2*	0.8 ± 0.4	1.1 ± 0.4	0.317
**Lipogenesis**			
*fasn*	1.1 ± 0.0	1.0 ± 0.1	0.081
*g6pd*	0.9 ± 0.1	0.9 ± 0.1	0.207
**Amino Acid Catabolism**			
*asat*	1.2 ± 0.1	0.7 ± 0.1	<0.001
*alat*	1.0 ± 0.1	0.9 ± 0.0	0.001
*gdh*	1.3 ± 0.6	0.8 ± 0.4	0.146
**Glucose Transport and Muscle Metabolism**			
*glut4*	0.7 ± 0.2	1.0 ± 0.3	0.022
*hk1*	1.0 ± 0.1	1.1 ± 0.1	0.005
*hk2*	0.9 ± 0.0	1.1 ± 0.1	0.011
*pfkma*	1.0 ± 0.1	0.9 ± 0.1	0.052
*pfkmb*	0.9 ± 0.2	0.9 ± 0.3	0.877
*pkma*	1.0 ± 0.1	1.1 ± 0.1	0.013

### Long-Term Effects of Early Glucose Stimuli on Growth Performance, Hepatic and Muscle Composition, Plasma Metabolites and Expression of Glucose Metabolic Genes in Juvenile Fish (20 Weeks)

**FIGURE 3:**
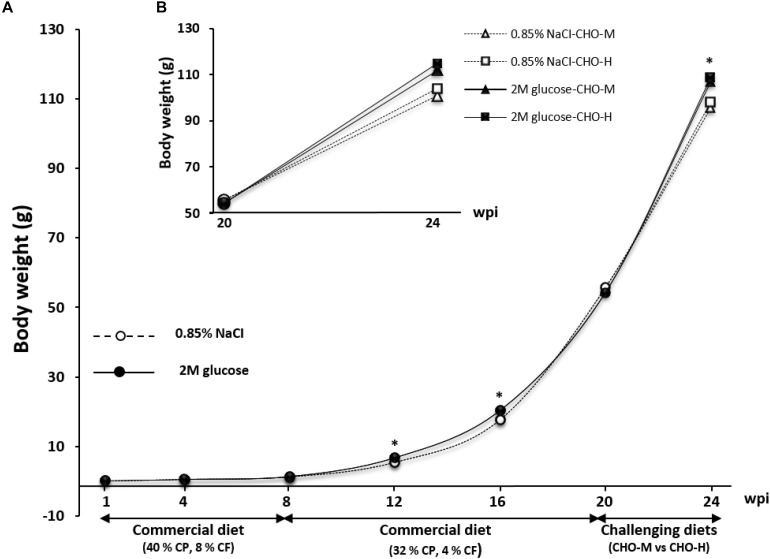
Growth response of fish injected with saline (0.85% NaCl) and glucose (2 M) during 1–24 wpi. Experimental fish were fed with commercial diets during 1–20 wpi. The asterisk indicates significant differences (*P* < 0.05) between fish injected with saline (0.85% NaCl) and glucose (2 M) at 12 and 16 wpi. The fish were then subjected to a challenge test with different dietary carbohydrate levels (37% carbohydrates, CHO-M; 67% carbohydrates, CHO-H) during weeks 21–24. Different growth responses were observed after the challenge test (24 wpi). **(A)** Whole experimental period. **(B)** Focus on the challenging period.

The growth performance of experimental fish until the juvenile stage is shown in Figure 3. Early glucose stimuli appeared to increase the growth performance of fish between weeks 12 and 16 (*P* < 0.05). At week 20, by contrast, there was no more significant difference in growth between the glucose-injected fish and normal-saline-injected fish. Note that a low water temperature (19–23°C) occurred during the culture period of weeks 16–20, which was lower than the optimal temperature for growth of Nile tilapia (28–30°C) (Qiang et al., 2014). This low temperature resulted in lower feed intake and consequently reduced the growth response in all experimental groups.

At week 20, early glucose stimuli led to an increase in the hepatic fat content in juvenile fish, while other chemical compositions, including protein, ash and glycogen, were not significantly different (Table 3). On the other hand, the hepatosomatic index (HSI) of both experimental fish appeared to be similar. While no significant differences between glucose-injected and normal-saline-injected fish were found in muscles for protein, fat and ash, early glucose injection led to increased glycogen content in the muscles (*P* < 0.05) (Table 3). This study also evaluated the effects of early glucose stimuli on the levels of plasma metabolites, such as glucose, triglycerides and BUN (Table 3). The levels of these plasma metabolites were similar between the experimental groups.

**TABLE 3:** Chemical composition and glycogen content in the liver and muscles, HSI and plasma metabolites of fish injected with saline (0.85% NaCl) and glucose (2 M) before (20 wpi) and after (24 wpi) challenging with CHO-M and CHO-H for 4 weeks (mean ± SD, *n* = 6).

Composition (g kg^–1^)	Before challenge			0.85% NaCl history		2 M glucose history		*P*-value^1^		
	0.85% NaCl history	2 M glucose history	*P*-value	CHO-M	CHO-H	CHO-M	CHO-H	History	Diet	Interaction
	
Chemical composition in liver at week 20				Chemical composition in liver at week 24						
Protein	102.5 ± 4.5	106.9 ± 5.0	0.134	106.5 ± 2.6^a^	95.63 ± 3.5^b^	99.5 ± 2.0^b^	72.7 ± 1.7^c^	< 0.001	<0.001	< 0.001
Fat	34.1 ± 3.7	39.4 ± 3.8	0.034	25.0 ± 1.0^c^	27.0 ± 0.7^b^	20.5 ± 0.9^d^	28.9 ± 1.0^a^	0.002	< 0.001	<0.001
Ash	9.6 ± 1.7	10.8 ± 0.7	0.148	11.2 ± 0.3	10.8 ± 0.6	11.5 ± 0.8	10.6 ± 1.7	0.896	0.146	0.530
Glycogen (mg g^–1^)	115.1 ± 9.7	108.8 ± 6.6	0.214	125.2 ± 24.5	207.8 ± 14.2	149.1 ± 34.3	236.4 ± 23.4	0.019	< 0.001	0.818
HSI^2^ (%)	1.7 ± 0.3	1.4 ± 0.3	0.086	3.0 ± 0.7^ab^	3.1 ± 1.3^ab^	4.2 ± 0.6^a^	2.6 ± 0.4^b^	0.299	0.038	0.022

**Chemical composition in muscles at week 20**				**Chemical composition in muscle at week 24**						

Protein	183.8 ± 2.0	184.4 ± 4.9	0.815	192.9 ± 7.4	192.4 ± 2.1	191.5 ± 1.8	189.2 ± 1.9	0.184	0.406	0.620
Fat	9.2 ± 0.6	9.1 ± 0.6	0.714	12.2 ± 0.8	12.3 ± 0.3	11.8 ± 0.9	12.4 ± 0.5	0.534	0.278	0.350
Ash	12.0 ± 0.2	11.9 ± 0.3	0.252	12.6 ± 0.3	12.7 ± 0.4	12.4 ± 0.1	12.5 ± 0.2	0.088	0.283	0.982
Glycogen (mg g^–1^)	4.0 ± 1.4	6.5 ± 1.4	0.010	3.7 ± 1.4	7.6 ± 2.6	5.8 ± 1.8	9.0 ± 1.9	0.048	< 0.001	0.652

**Plasma metabolites at week 20**				**Plasma metabolites at week 24**						

Glucose (mM)	4.5 ± 0.5	4.6 ± 0.6	0.742	4.6 ± 0.4	4.7 ± 0.3	5.0 ± 0.6	5.5 ± 0.5	0.005	0.185	0.257
Triglycerides (mM)	1.5 ± 0.2	1.4 ± 0.4	0.451	2.3 ± 0.7	3.9 ± 0.3	3.0 ± 0.7	4.0 ± 0.6	0.086	< 0.001	0.256
BUN^3^ (mM)	1.03 ± 0.06	0.91 ± 0.20	0.206	0.94 ± 0.14	0.91 ± 0.08	0.85 ± 0.07	0.79 ± 0.10	0.020	0.302	0.828

*^1^Two-way ANOVA was used to analyze the effects of glucose injection (History), challenging diet (Diet) and their interaction (History × Diet). Different letters indicate significant differences in the mean values for four combination groups (P < 0.05). ^2^HSI = 100 × (liver weight/body weight). ^3^BUN, blood urea nitrogen.*

**FIGURE 4:**
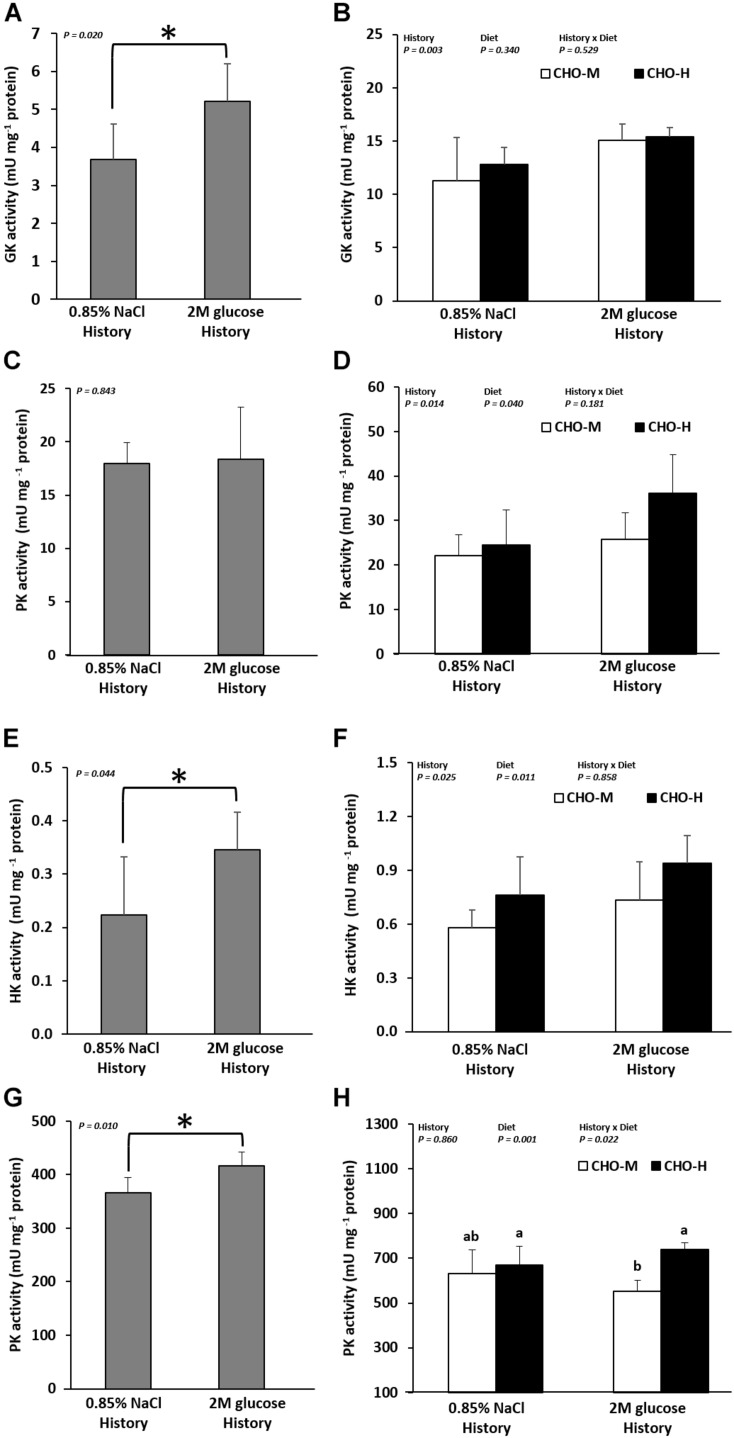
Enzyme activity (mU mg^–1^ protein) in the livers and muscles of Nile tilapia that were microinjected with either 0.85% NaCl or 2 M glucose (history). GK **(A,B)** and PK **(C,D)**, which are involved in glycolysis, were analyzed in the liver samples. HK **(E,F)** and PK **(G,H)**, which are involved in glycolysis, were analyzed in the muscle tissue samples. At 21–24 wpi, the fish were subjected to a challenge test with different dietary carbohydrate levels (37% carbohydrates, CHO-M; 67% carbohydrates, CHO-H). Before the challenge test, the fish were sampled to determine the effect of the glucose injection history on the GK and PK activity in the liver **(A,C)** and the HK and PK activity in the muscles **(E,G)**. The asterisk in the bar graph indicates a significant difference (*P* < 0.05). After the challenge test (24 wpi), the combination effects of glucose history and challenging diet on the GK and PK activity in the liver **(B,D)** and the HK and PK activity in the muscles **(F,H)** were examined. Data are presented as the mean ± standard deviation (SD) (*n* = 6). Two-way ANOVA was used to analyze the effects of glucose injection (History), challenging diet (Diet) and their interaction (History × Diet). When significant interaction effects were observed, one-way ANOVA following Tukey’s range test was performed to rank the treatment combination groups. Different letters in the bar graph indicate significant differences (*P* < 0.05).

The long-term effects of early glucose stimuli on the expression of genes related to glucose metabolism in muscles and livers were also shown in fish at 20 weeks (Table 4 and Figure 4). The results showed that early glucose stimuli led in the long term to higher expression of hepatic GK (both *gck* mRNA levels and enzyme activity) (Figure 4A) (*P* < 0.05). Muscle HK1 and HK2 (both *hk1* and *hk2* mRNA levels and enzyme activity) (Figure 4E) and PK (enzyme activity) (Figure 4G) were also higher in fish with an early glucose stimulus history (*P* < 0.05). However, we can observe that the expressions of other genes related in glucose metabolic pathways in the liver and muscles (*pfklr*, *pklr*, *g6pca1*, *g6pca2*, *pk1*, *pck2*, *fasn*, *g6pd*, *asat*, *alat*, *gdh*, *glut4*, *pfkma*, *pfkmb*, and *pkma*) and hepatic PK (enzyme activity) (Figure 4C) were not significantly different (*P* > 0.05).

**TABLE 4:** mRNA levels of genes involved in carbohydrate metabolism in the liver and muscles of fish injected with saline (0.85% NaCl) and glucose (2 M) before (20 wpi) and after (24 wpi) challenging with CHO-M and CHO-H for 4 weeks (mean ± SD, *n* = 6).

Genes	Before challenge			0.85% NaCl history		2 M glucose history		*P*-value^1^		
	0.85% NaCl history	2 M glucose history	*P*-value	CHO-M	CHO-H	CHO-M	CHO-H	History	Diet	Interaction
	
Liver glycolysis at week 20				Liver glycolysis at week 24						
*gck*	0.4 ± 0.3	0.8 ± 0.2	0.027	0.5 ± 0.2	1.0 ± 0.3	1.3 ± 1.0	2.4 ± 0.8	0.001	0.010	0.329
*pfklr*	1.0 ± 1.0	0.9 ± 0.4	0.810	1.4 ± 0.9	1.8 ± 1.4	2.6 ± 2.2	2.0 ± 1.9	0.314	0.889	0.485
*pklr*	1.4 ± 1.4	0.8 ± 0.3	0.325	1.0 ± 0.2	1.1 ± 0.7	1.3 ± 0.5	1.6 ± 0.3	0.024	0.360	0.655

**Liver gluconeogenesis at week 20**				**Liver gluconeogenesis at week 24**						

*g6pca1*	1.3 ± 0.5	1.4 ± 0.8	0.626	1.3 ± 0.5	0.9 ± 0.4	1.1 ± 0.4	0.8 ± 0.1	0.313	0.059	0.715
*g6pca2*	0.9 ± 0.3	1.2 ± 0.4	0.103	1.6 ± 0.9	0.9 ± 0.4	1.4 ± 0.5	0.8 ± 0.5	0.856	0.003	0.477
*pck1*	0.6 ± 0.4	1.4 ± 1.3	0.230	1.5 ± 0.9	0.8 ± 0.5	0.8 ± 0.6	0.2 ± 0.1	0.016	0.010	0.779
*pck2*	0.7 ± 0.3	0.7 ± 0.6	0.938	2.9 ± 1.8	0.6 ± 0.6	1.7 ± 1.4	0.7 ± 0.4	0.244	0.003	0.209

**Liver lipogenesis at week 20**				**Liver lipogenesis at week 24**						

*fasn*	1.2 ± 1.0	1.0 ± 0.3	0.682	1.1 ± 0.8	1.3 ± 0.8	1.1 ± 0.8	2.3 ± 0.2	0.087	0.031	0.111
*g6pd*	0.9 ± 0.5	1.3 ± 0.7	0.328	0.9 ± 0.4	1.4 ± 0.7	0.9 ± 0.3	2.4 ± 1.2	0.077	0.007	0.211

**Liver amino acid catabolism at week 20**				**Liver amino acid catabolism at week 24**						

*asat*	0.9 ± 0.5	1.3 ± 0.5	0.266	1.4 ± 0.5^b^	0.4 ± 0.2^c^	2.1 ± 0.5^a^	0.2 ± 0.1^c^	0.119	< 0.001	0.010
*alat*	0.9 ± 0.4	1.1 ± 0.2	0.280	2.3 ± 1.6	1.1 ± 0.7	2.7 ± 1.3	1.0 ± 0.4	0.810	0.005	0.651
*gdh*	1.0 ± 0.5	1.2 ± 0.2	0.470	1.7 ± 0.5	1.2 ± 0.4	1.7 ± 0.7	1.3 ± 0.3	0.937	0.039	0.904

**Glucose transport and muscle metabolism at week 20**				**Glucose transport and muscle metabolism at week 24**						

*glut4*	0.8 ± 0.4	1.2 ± 0.3	0.071	0.6 ± 0.4	0.8 ± 0.1	1.1 ± 0.3	1.1 ± 0.4	0.005	0.327	0.631
*hk1*	0.8 ± 0.3	1.2 ± 0.3	0.047	0.7 ± 0.2	0.9 ± 0.2	0.9 ± 0.2	1.2 ± 0.2	0.019	0.018	0.656
*hk2*	0.7 ± 0.3	1.1 ± 0.3	0.030	0.8 ± 0.2	0.9 ± 0.4	1.1 ± 0.3	1.2 ± 0.1	0.009	0.225	0.752
*pfkma*	0.8 ± 0.4	1.2 ± 0.7	0.262	1.0 ± 1.0	0.7 ± 0.2	0.7 ± 0.3	0.9 ± 0.5	0.853	0.825	0.305
*pfkmb*	1.1 ± 0.2	0.8 ± 0.3	0.165	1.2 ± 0.6	0.9 ± 0.3	1.0 ± 0.3	1.1 ± 0.2	0.971	0.407	0.237
*pkma*	0.8 ± 0.3	1.0 ± 0.4	0.617	0.8 ± 0.1^b^	0.8 ± 0.2^b^	0.7 ± 0.1^b^	1.1 ± 0.2^a^	0.205	0.002	0.015

*^1^Two-way ANOVA was used to analyze the effects of glucose injection (History), challenging diet (Diet) and their interaction (History × Diet). Different letters indicate significant differences in the mean values for four combination groups (P < 0.05).*

### Combination Long-Term Effects of Early Glucose Stimuli and 4-Week High-Dietary-Carbohydrate Challenge in Fish at 24 Weeks

The effects of early glucose stimuli combined with a challenge with a high-carbohydrate diet were examined in normal-saline-injected and glucose-injected fish by feeding them CHO-M and CHO-H diets during weeks 21–24 (Figure 1). Table 5 shows that, irrespective of the diet, the glucose injection history led to a significant improvement in the growth performance in terms of the final body weight, average daily gain, specific growth rate and feed conversion ratio (FCR) (*P* < 0.05). By contrast, no significant effects of the two diets on growth performance were observed (*P* > 0.05).

**TABLE 5:** Growth performance of fish injected with saline (0.85% NaCl) and glucose (2 M) at 24 wpi after challenging with CHO-M and CHO-H for 4 weeks (mean ± SD, *n* = 6).

	0.85% NaCl history		2 M glucose history		*P*-value^a^		
	CHO-M	CHO-H	CHO-M	CHO-H	History	Diet	Interaction
Initial weight (g)	54.3 ± 0.7	54.7 ± 0.4	54.7 ± 0.6	55.1 ± 0.4	0.079	0.073	0.875
Final weight (g)	107.4 ± 2.0	108.9 ± 5.8	114.8 ± 5.5	116.0 ± 4.7	0.001	0.492	0.931
ADG^b^ (g day^–1^)	1.8 ± 0.1	1.8 ± 0.2	2.0 ± 0.2	2.0 ± 0.2	0.002	0.613	0.948
SGR^c^ (% day^–1^)	2.3 ± 0.1	2.3 ± 0.2	2.5 ± 0.1	2.5 ± 0.1	0.002	0.787	0.976
FI^d^ (% day^–1^)	2.4 ± 0.1	2.4 ± 0.1	2.5 ± 0.2	2.4 ± 0.1	0.655	0.459	0.459
FCR^e^	1.2 ± 0.1	1.1 ± 0.1	1.0 ± 0.1	1.0 ± 0.1	0.013	0.201	0.781
Survival rate (%)	98.3 ± 4.1	100.0 ± 0.0	96.7 ± 8.2	100.0 ± 0.0	0.660	0.195	0.660

*^a^Two-way ANOVA was used to analyze the effects of glucose injection (History), challenging diet (Diet) and their interaction (History × Diet). ^b^Average daily gain (ADG) = (final body weight - initial body weight)/experimental days. ^c^Specific growth rate (SGR) = 100 × [(ln final body weight - ln initial body weight)/experimental days]. ^d^Feed intake (FI) = 100 × (dry feed intake/[(initial wet body mass + final wet body mass)/2])/days. ^e^Feed conversion ratio (FCR) = dry feed fed/wet weight gain.*

The effects of glucose history as well as those of the two diets on the chemical composition in the liver and muscles are shown in Table 3. Early glucose injection stimuli together with dietary carbohydrates led to a decrease in the protein content in the liver (*P* < 0.05). Interaction effects were also observed, showing that the glucose-injected fish that were fed with CHO-H had the lowest hepatic protein level (*P* < 0.05). Both early glucose stimuli and challenging diets had significant effects on the hepatic lipid and glycogen content (*P* < 0.05). The glucose-injected fish that were fed with CHO-H had the highest liver fat and glycogen level (*P* < 0.05). Dietary carbohydrates had an effect on the hepatosomatic index, and its interaction effect with glucose stimuli was found. Moreover, the glucose-injected fish fed with CHO-H had the lowest HSI (*P* < 0.05). Regarding the muscle composition, both glucose history and dietary carbohydrates were observed to have a significant effect only for glycogen (*P* < 0.05) (Table 3). Note that glucose injection history and high dietary challenge led to modulate the protein content in Nile tilapia carcass (*P* < 0.05) (data not shown). Both glucose history and high dietary challenge resulted in an increase in the glycogen content in muscles (*P* < 0.05). Again, the glucose-injected fish fed with CHO-H had the highest glycogen content in the muscles compared to other groups.

Glucose history had a significant effect on plasma glucose and BUN and was associated with an increase in plasma glucose and a decrease in BUN (Table 3) (*P* < 0.05). Only the effects of challenging diet on plasma triglycerides were observed (Table 3) (*P* < 0.05).

Table 4 shows the effects of the glucose stimulus history and dietary challenge on the expression of glucose metabolic genes. The glucose stimulus history is associated with the up-regulation of *gck*, *pklr*, *hk1*, *hk2*, and *glut4*; indeed, the expression of *gck*, *pklr*, *hk1*, and *hk2* was higher at both mRNA and enzymatic levels (*P* < 0.05) (Table 4 and Figures 4A,D,F). In addition, fish with a glucose history had lower *pck1* mRNA levels. Moreover, no significant differences were observed for *pfklr*, *g6paca1*, *g6pca2*, *pck2*, *fasn*, *g6pd*, *asat*, *alat*, *gdh*, *pfkma*, *pfkmb*, and *pkma* (*P* > 0.05). Fish fed with a high-carbohydrate diet exhibited higher expression of *gck* (mRNA level), *pk* (enzyme activity), *hk1* (mRNA level and enzyme activity), and *hk2* (enzyme activity) (*P* < 0.05) (Table 4 and Figures 4D,F,H). Furthermore, dietary CHO-H down-regulated all the examined genes in gluconeogenesis (except for *g6pca1*), lipogenesis and amino acid catabolism in the liver (*P* < 0.05) (Table 4). It can be noticed that there was an interaction effect between the glucose stimuli and dietary carbohydrates for the *asat* and *pkma* genes, showing that glucose-injected fish fed with CHO-H had the lowest hepatic *asat* and highest muscle *pkma* gene expression (Table 4).

**FIGURE 5:**
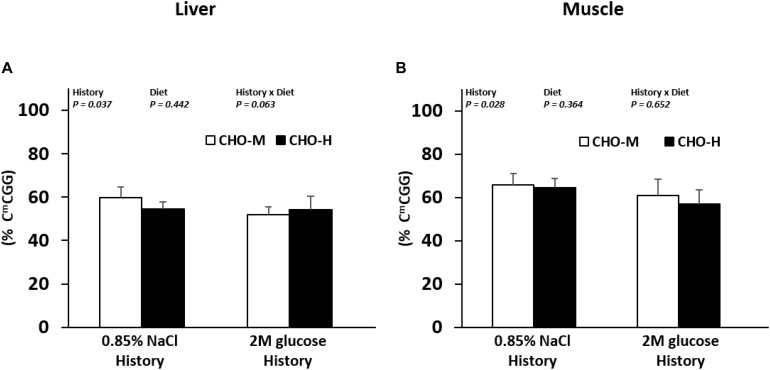
Effects of the glucose injection history and dietary carbohydrate challenge on the global DNA methylation in Nile tilapia. The levels of 5-methylcytosine (5-mC%: 5-mC/total DNA) in the DNA in the liver **(A)** and muscles **(B)** were measured in fish challenged with different dietary carbohydrate levels (37% carbohydrates, CHO-M; 67% carbohydrates, CHO-H). Data are presented as the mean ± SD (*n* = 6). Two-way ANOVA was used to analyze the effects of glucose injection (History), challenging diet (Diet) and their interaction (History × Diet).

Because the mechanisms that can be at the origin of programming (hereby glucose history) could be mediated by epigenetic modifications, we investigated the effects of the glucose history (and also dietary carbohydrate diets) on global DNA methylation using a LUMA approach. Fish with a glucose stimulus history showed a lower methylation level at C^m^CGG in the liver and muscle compared to the control fish, whereas the dietary carbohydrate challenge had no effect on this parameter (Figure 5).

## Discussion

Recently, it has been revealed that early nutritional programming exerts several modulating effects on metabolic processes in juvenile/adult fish, making this a promising approach for fish nutrition (Panserat et al., 2019). Particularly, the effects of nutritional programming on carbohydrate metabolism have been intensively studied in aquaculture fish with a low capacity to efficiently use carbohydrates (Geurden et al., 2007, 2014; Fang et al., 2014; Rocha et al., 2014, 2015, 2016a,b; Gong et al., 2015; Liang et al., 2017). Among vertebrates, fish exhibit various feeding habits. Therefore, in order to provide a general and comparative concept of nutritional programming in fish, a deeper investigation of the effects of nutritional programming in omnivorous fish, which are capable of using carbohydrates as a primary energy source, is required. Nile tilapia have been known to be a good user of dietary carbohydrates, and their ability to adapt to a high-carbohydrate dietary level has been well demonstrated (Shiau and Peng, 1993; Wang et al., 2005; Gaye-Siessegger et al., 2006; Azaza et al., 2015; Boonanuntanasarn et al., 2018a, b). During very early development, nutrient intake of fish is relied only on their yolk reserves, and ontogenic development of digestive tract and their relative organs are ongoing (Morrison et al., 2001). Indeed, the prenatal embryonal stage was demonstrated to be an early developmental stage for efficient nutritional programming in mammals (reviewed by Langley-Evans, 2009). This is why we chose the larval stage to inject glucose into the yolk reserves during the very early development stage. Our findings demonstrated for the first time the existence of nutritional programming in Nile tilapia, a model of omnivorous fish.

### Direct Effects of Early Glucose Stimuli on the Survival and Glucose Metabolic Gene Expression of Alevins

Nutritional intervention at early developmental stages is an important factor for recording the programming stimuli that contribute to persistent effects during the later lifespan (Lucas, 1998). Up till now, most nutritional interventions to induce nutritional programming have been conducted during the first feeding stage in fry (Geurden et al., 2007, 2014; Fang et al., 2014; Gong et al., 2015; Rocha et al., 2016a, b; Liang et al., 2017). In the present study, we conducted an early nutritional stimulus through the microinjection of glucose into the yolk sacs of Nile tilapia alevins at stage 17 (Fujimura and Okada, 2007). Our results showed that the direct survival and growth rates of fish injected with 2 M glucose or with the saline solution were similar, suggesting that injecting 2 M glucose did not cause any negative effects on the fish. In this study, an increase in the glucose level in the yolk revealed the successful of glucose receive in fish alevins, which also led to an increase in the glycogen and glucose content in glucose-injected larvae at 1 wpi. These findings demonstrated that the injected glucose was efficiently extracted from the yolk to the animal and subsequently converted into glycogen deposition in the larvae.

The effects of early glucose stimuli on the glucose-metabolism-related gene expression were assessed in larvae at 1 wpi, which is assumed to be the stage of almost complete absorption of the injected glucose from the vitellus to the larvae. Our results showed that, at the transcriptional level, glucose stimuli during the early alevin stage exerted the expected effects on carbohydrate-metabolism-related pathways (Geurden et al., 2014). Indeed, the increase in the expression of hepatic *pklr* and muscular *hk1*, *hk2* and *pkma* together with *glut4* (mRNA) implies that the fish received well the glucose stimulus at the molecular level. In the same way, the glucose stimuli down-regulated the expression of several enzymes related to gluconeogenesis (*g6pca1*, *g6pca2*, and *pck1*) and amino acid catabolism (*asat*, *alat*) as expected (Geurden et al., 2014). There are few reports describing the effects of hyperglucidic stimuli via glucose injection into the yolk sac. Similar to our findings, in zebrafish, microinjection of glucose into yolk at 0.2 dpf (30% epiboly stage) resulted in the down-regulation of gluconeogenic genes (*g6pca1*, *pck 2*) in larvae (Rocha et al., 2014, 2015). However, the glucose stimuli suppressed the expression of the muscular glycolytic gene (*pkma*), whereas other genes in the hepatic and muscular glycolysis pathways remained unchanged, which is different from the tilapia in the present study. Our data suggest strong direct effects of glucose injection on the regulation of glucose metabolism. It was also possible to test the existence of metabolic programming 20 weeks and 24 weeks after the early stimuli.

### Long-Term Effects of Early Glucose Stimuli on Growth Performance and Glucose Metabolism in Juvenile Fish Fed With a Commercial Diet

In the present study, the glucose history during the alevin stage led to an increase in the growth performance of Nile tilapia until the fingerling stage (a significant improvement was observed at 12–16 wpi). Subsequently, normal-saline-injected fish showed catch-up growth (at 20 wpi) when compared to hyperglucidic stimuli fish. This is similar to what was previously observed regarding the maternal effects in fish: it was demonstrated that maternal/parental effects resulted in faster growth only during a short period of the early life of the descendants, and compensatory mechanisms were exhibited later (Heath et al., 1999; Donelson et al., 2009).

Moreover, our data demonstrated the long-term effects of glucose overload in the yolk sac on several metabolic actors in the liver and muscles of juvenile fish. Indeed, this study showed that the early glucose stimulus during the early alevin stage persisted up to 20 weeks in the carbohydrate-metabolism-related pathways (even though these changes were less pronounced than those observed directly after glucose injection). Indeed, our findings revealed that glucose stimuli led to the induction of glycolysis pathways in the liver (*gck*, both mRNA level and enzyme activity) and muscles (both mRNA level of *hk1* and *hk2* and their enzyme activity), as well as muscular PK activity in the long term. In addition, a higher muscle glycogen content in glucose-injected fish was detected, confirming that the whole glucose metabolism process was modified in juvenile tilapia by the early glucose stimulus. It must also be noted that although there were no variations in lipogenic gene mRNA levels, there was an increase in the juvenile hepatic fat content of glucose-injected fish. Combined together, early glucose stimuli exerted long-term effects on carbohydrate (and probably lipid) metabolism in juvenile fish and demonstrated clear metabolic programming in tilapia for the first time.

Finally, the higher expression of the glycolytic pathway suggests that the early glucose stimulus led to an increase in the use of glucose as an energy source in Nile tilapia (even though tilapia are well known to be a better user of dietary carbohydrates compared to carnivorous fish) (Schrama et al., 2012, 2018; Polakof et al., 2012; Boonanuntanasarn et al., 2018a, b). Programming of glucose metabolism seems to be associated with a better growth performance between 12 and 16 wpi, suggesting that the early glucose injection could be associated with a better use of carbohydrates, leading to a higher dietary protein-sparing effect.

### Long-Term Effects of Early Glucose Injection on Growth Performance and Glucose Metabolism in Juvenile Fish When Fed With Different Levels of Carbohydrates

Our data clearly showed a better growth performance in fish previously injected with glucose after challenging with the two diets (weeks 21–24), demonstrating that early glucose stimuli promoted protein sparing by dietary carbohydrates in the juvenile stage, as suggested also during the 12–16 wpi stage (see above). These findings suggested that hyperglucidic stimuli during the alevin stage have positive effects on the growth performance later in life. By contrast, glucose injection into the yolk sac of zebrafish (*Danio rerio*) was not associated with a better growth performance in juvenile fish (Rocha et al., 2014, 2015), suggesting that the effects of glucose injection depend on the fish species.

The combination effects of early hyperglucidic stimuli and high-carbohydrate diet on the biochemical composition, liver size, muscle composition and plasma metabolites were shown to be an important line of evidence to reveal the existence of nutritional programming. First, high-carbohydrate diets exerted expected effects on the chemical composition of the liver and muscles: (1) reduced protein in the liver, (2) increased fat in the liver and (3) elevated glycogen in the liver and muscles. Indeed, the effects of high-carbohydrate dietary intake were similar to those in previous reports (Azaza et al., 2015; Wang et al., 2017; Boonanuntanasarn et al., 2018a). Combined with the effects of early glucose stimuli, our results suggested that the hyperglucidic stimulus history modulated the effects of high-carbohydrate diet on the liver composition: early glucose stimuli and high-carbohydrate diets synergistically reduced hepatic proteins, increased hepatic lipids and increased glycogen in the liver and muscles. Moreover, regarding the plasma metabolites, as expected, there was an increase in plasma triglycerides (in relation to the higher fat content of the tissues), however, in contrast to previous studies (Boonanuntanasarn et al., 2018a, b), no variations in glycemia or BUN were detected with carbohydrate intake. While the effect of the glucose stimulus history on plasma metabolites was not observed in fish at 20 wpi, after the dietary challenge, there was an increase in glycemia (even though at a relatively low level, i.e., 5.5 mM maximum) and a decrease in BUN in glucose-injected fish. Lower levels of plasma BUN in glucose-injected fish could indicate lower amino acid catabolism, which can be related to a better growth performance in these fish. Taken together, the glucose stimulus history exerted modulating effects on biochemical parameters (nutrient composition, plasma metabolites) later in life. It would be very interesting if these metabolic effects can be related to specific regulation of enzymes, in particular at the molecular level, as expected in the context of programming (Symonds et al., 2009).

First, as shown in previous studies (Boonanuntanasarn et al., 2018a, b), our results showed that the increase of dietary carbohydrates and the concomitant decrease of dietary proteins are associated with (1) an increase in *gck*/*hk1* (glucose phosphorylation) and *fasn-g6pd* (lipogenesis) and (2) a decrease in *g6pca1*, *pck1*, and *pck2* (gluconeogenesis) and in *asat*, *alat* and *gdh* (amino acid catabolism). Moreover, the high glycolytic activity for PK (in the liver and muscles) and HK (muscle) confirmed the good adaptation of tilapia to carbohydrates (Wang et al., 2005, 2017; Figueiredo-Silva et al., 2013; Boonanuntanasarn et al., 2018a, b). The most important and original point here is that early glucose stimuli appeared to synergistically influence liver glycolysis and gluconeogenesis and muscle glucose metabolism. Indeed, glycolytic enzymes (*gck* expression and its enzyme activity, *pk* expression and its enzyme activity) as well as muscle *glut4* (mRNA) and *hk1*/*2* (mRNA and enzyme activity) were higher in glucose-injected tilapia. In zebrafish injected with glucose in the yolk, fish at 1 dpf fed with a high-carbohydrate diet showed up-regulated metabolic marker genes in muscle glycolysis (*hk1* and *6pfk*) and down-regulated gluconeogenesis as in tilapia, whereas almost no more effects were observed in juvenile fish (Rocha et al., 2014, 2015). Combined together, these findings strongly suggest that alevins receiving glucose injection could remember it and subsequently influence (positively) glucose metabolism in juvenile fish.

Overall, our findings revealed the existence of nutritional programming of metabolism in juvenile tilapia. We, therefore, investigated whether the modulation of metabolism could be linked to a modification in the global epigenetic landscape which is a potential mechanism for long-lasting modulation of metabolic gene expression (Symonds et al., 2009). Carbohydrate diets did not exert an effect on the global DNA methylation in the liver and muscles. Dietary carbohydrate intake was previously associated with total DNA hypomethylation in both the liver and muscles of rainbow trouts (Craig and Moon, 2013; Marandel et al., 2016a) when compared with fish fed without carbohydrates both in the short (4 days of nutrition; Marandel et al., 2016a) and the long (eight weeks of nutrition; Craig and Moon, 2013) term. However, compared to the present study, in which we searched for an estimation of the global DNA methylation only at C^m^CGG sites using the LUMA technique, these authors evaluated the genomic DNA methylation level in an exhaustive way using an enzyme-linked immunoscorbent assay technique. Thus, regarding our results, we could not exclude the fact that DNA methylation may occur at non-CCGG sites (i.e., other CpG sites or non-CpG sites), sites not monitored with the LUMA assay. Moreover, previous studies were performed on rainbow trouts, whose metabolism and physiology are quite different from those of Nile tilapia. DNA methylation/demethylation pathways may, thus, differ between these two species, leading to different results. On the other hand, interestingly, in juvenile fish with early glucose injection, we found global C^m^CGG hypomethylation in the liver and muscles compared to non-injected fish. Our data suggest that early nutritional stimuli could have a long-term effect on the DNA methylation of the genome of tilapia, as observed previously in juvenile rainbow trouts after first feeding with high levels of vitamins (Panserat et al., 2017).

In conclusion, early glucose stimuli in the larval stage led to an impressive improved growth performance in Nile tilapia. This could be associated with permanent up-regulation of glycolysis and glucose transporters and down-regulation of gluconeogenesis and amino acid catabolism in glucose-injected fish, suggesting a better use of carbohydrates as an energy source and a protein-sparing effect. The glucose stimulus history is more pronounced when the fish are challenged with medium- and high-carbohydrate diets. The mechanisms at the origin of this programming could be due to at least an epigenetic mechanism, as revealed by the global DNA hypomethylation in the liver and muscles of juvenile fish.

## Data Availability Statement

All datasets generated for this study are included in the article/Supplementary Material.

## Ethics Statement

All experimental protocols were approved by the Ethics Committee of Suranaree University of Technology, Animal Care and Use Committee (Approval no. A-18/2562).

## Author Contributions

SB and SP conceptualized research idea and directed the experimental design. SK performed diet preparation, fish culture experiment, and statistical analysis. EP-J and VV contributed experiments for gene expression and enzymatic assays. All authors contributed preparation of manuscript. All authors have read an approved the final manuscript.

## Conflict of Interest

The authors declare that the research was conducted in the absence of any commercial or financial relationships that could be construed as a potential conflict of interest.
