# Supplementary material for: Glucose Injection Into Yolk Positively Modulates Intermediary Metabolism and Growth Performance in Juvenile Nile Tilapia (Oreochromis niloticus)
Source: Front Physiol. 2020 Apr 17;11:286. doi: 10.3389/fphys.2020.00286 (PMC7181793; doi:10.3389/fphys.2020.00286)
Supplement: Supplementary file 1 [file Table_1.pdf]

Supplementary Table S1. List of tilapia primers used for qRT-PCR.

Genes	5′/3′ forward primer	5′/3′ reverse primer	Access number
Reference gene			
*ef1**	GCACGCTCTGCTGGCCTTT	GCGCTCAATCTTCCATCCC	AB075952
Liver metabolism			
gck	GGGTGGTAGGATTTGGTGTG	TGCTGACACAAGGCATCTTC	XM__003451020
pfklr	GACGAGCGAGTGGAGAAAAC	TGTCTTGATCCGAGGGAATC	XM__003447353
pklr	AGGTACAGGTCACCCGTCAG	CATGTCGCCAGACTTGAAGA	XM__005472622
g6pca1	AGCGTTAAGGCAACTGGAGA	AAAAGCTAACAAGGCCAGCA	XM__003448671
g6pca2	CTTCTTCCCCCTTTGGTTTC	AGACTCCTGCAGCTCCCATA	XM__013273429
pck1	AAGCTTTTGACTGGCAGCAT	TGCTCAGCCAGTGAGAGAGA	XM__003448375
pck2	TACGTCTTGAGCTCCCGTCT	CCTCCTGGATGATGCAAGTT	XM__019354843
fasn	AACCTGCTTCTCAAGCCAAA	CGTCACCCCTTGTTCTTTGT	XM__013276809
g6pd	GTCACCTCAACCGGGAAGTA	TGGCTGAGGACACCTCTCTT	XM__013275693
asat	GCTTCCTTGGTGACTTGGAA	CCAGGCATCTTTCTCCAGAC	XM__003451918
alat	CACGGTGAAGAAGGTGGAGT	GCAGTTCAGGGTAGGAGCAG	XM__005476466
gdh	CGAGCGAGACTCCAACTACC	TGGCTGTTCTCATGATTTGC	XM__003457465
Muscle metabolism			
glut4	GAGGATGGACATGGAGAGGA	CAGGAAAAGCGAGACTACCG	JN900493
hk1	CGTCGCTTAGTCCCAGACTC	TGACTGTAGCGTCCTTGTGG	XM_019360229
hk2	CAGAGGGGAATTCGATTTGA	CCCACTCGACATTGACACAC	XM_003448615
pfkma	AGGACCTCCAACCAACTGTG	TTTTCTCCTCCATCCACCAG	XM_019349871
pfkmb	TTTGTGCATGAGGGTTACCA	CACCTCCAATCACACACAGG	XM_003441476
pkma	TGACTGCTTCCTGGTCTGTG	CAGTGAAAGCTGGCAAATGA	XM_005447626

[*]From Yang et al. (2013).
